# Effects of Roasting Process on Sensory Qualities, Color, Physicochemical Components, and Identification of Key Aroma Compounds in Hubei Strip-Shaped Green Tea

**DOI:** 10.3390/metabo15030155

**Published:** 2025-02-25

**Authors:** Fei Ye, Anhui Gui, Xiaoyan Qiao, Panpan Liu, Xueping Wang, Shengpeng Wang, Lin Feng, Jin Teng, Jinjin Xue, Xun Chen, Yuanhong Mei, Binghua Zhang, Hanshan Han, Anhua Liao, Pengcheng Zheng, Shiwei Gao

**Affiliations:** 1 Fruit and Tea Research Institute, Hubei Academy of Agricultural Sciences, Wuhan 430064, China; yf421@hbaas.com (F.Y.);; 2 Guangdong Provincial Key Laboratory of Tea Plant Resources Innovation and Utilization, Tea Research Institute, Guangdong Academy of Agricultural Sciences, Guangzhou 510610, China; 3 Hubei Wufeng Jiming Tea Co., Ltd., Wufeng County, Yichang 443413, China; 4 Danding Tea Co., Ltd., Danjiangkou County, Shiyan 442717, China; 5 MuLan Tianxiang Co., Ltd., Huangpi District, Wuhan 432200, China; 6 Huaishu Tea Professional Cooperative, Yunxi County, Shiyan 442616, China

**Keywords:** Hubei strip-shaped green tea, roasting process, color, physicochemical components, aroma quantitation, odor activity value, HS-SPME-GC-MS-O

## Abstract

Background: Roasting conditions significantly influence the sensory profile of Hubei strip-shaped green tea (HSSGT). Methods: This study examined the effects of roast processing on the sensory attributes, color qualities, physicochemical properties, and key aroma compounds of HSSGT. Sensory evaluation, color qualities determination, principal component analysis of physicochemical components (PCA), HS-SPME (headspace solid-phase microextraction) coupled with GC-MS (gas chromatography–mass spectrometry), relative odor activity value (ROAV), gas chromatography–olfactometry (GC-O), and absolute quantification analysis were employed to identify the critical difference in compounds that influence HSSGT desirability. Results: The results indicated that HSSGT roasted at 110 °C for 14 min achieved the highest sensory scores, superior physicochemical qualities, and an enhanced aroma index, which was attributed to shifting the proportion of chestnut to floral volatile compounds. Additionally, sensory-guided ROAV, GC-O, and absolute quantification revealed that linalool, octanal, nonanal, and hexanal were the most significant volatile compounds. The variations in these four critical compounds throughout the roasting process were further elucidated, showing that the ideal roasting conditions heightened floral aromas while diminishing the presence of less desirable green odors. These findings offer technical guidance and theoretical support for producing HSSGT with a more desirable balance of chestnut and floral aroma characteristics.

## 1. Introduction

Green tea, renowned for its pleasurable taste and health advantages, is a highly popular beverage worldwide [1]. Green tea dominates the Chinese tea market, representing 60% of total sales. It is generally classified into four types based on shape: flat, curled, round, and strip-shaped. Strip-shaped green tea (SSGT) is renowned for its tight/straight shape, vibrant yellow-green infusion, crisp and robust aroma, and refreshing, smooth taste [2,3,4]. Hubei strip-shaped green tea (HSSGT) includes a wide variety of teas from different Hubei Province regions, such as Enshi Yulu from the Enshi region [4], Yichang Maojian from the Yichang region, Wudang Mountain tea from the Shiyan region, Xiangyang high-aroma tea from the Xiangyang region, and Jinshui Cuifeng from the Wuhan region. According to the Hubei Tea Association, HSSGT accounts for more than 30% of the total output of Hubei green tea, while the domestic market sales share accounts for more than 50%.

The color and taste were important factors of tea qualities and were related to the physicochemical qualities of strip green teas [2], such as the tea polyphenols, free amino acids, soluble sugars, caffeine, and catechins, which are also the material basis for health benefits of tea [1]. Determining the color and physicochemical qualities of HSSGT roasted with different processes would improve the flavor of tea and increase the HSSGT prices. Green tea’s aroma is a vital component of its sensory attributes, and it plays a crucial role in determining its sensory quality and market price [5,6]. Tea aroma is significantly influenced by the processing techniques used. Traditional HSSGT processing typically involves spreading, fixation, shaping, drying, and roasting. Spreading and fixation are important for the formation of a high-quality aroma of green tea, while shaping, including the rolling or re-rolling process, is key for the formation of the tight and straight appearance of strip green tea. Roasting is critical for developing chestnut-like or intense, high aromas and a mature flavor [7,8,9,10]. However, roasting parameters, particularly temperature and time, are often determined empirically and lack a systematic research approach. As the market for HSSGT grows, it is crucial to optimize roasting parameters for large-scale machine processing to ensure a consistent, high-quality flavor profile, and enhanced aroma with minimized bitterness and astringency [11].

HS-SPME-GC-MS are essential tools for studying the characteristics and formation mechanisms of tea aroma quality. While many aroma compounds present in low concentrations can significantly influence the overall aroma profile, compounds with ROAV values exceeding one are considered to contribute more prominently to the perceived aroma. For example, previous research using HS-SPME-GC-MS combined with ROAV analysis has shown that floral and fruity notes in tea are mainly attributed to alcohols [12], while some researchers using GC-MS platforms found that the moderate rubbing degree was beneficial to the shape and aroma formation of strip green tea [5].

To date, no systematic scientific investigations have explored the impact of roasting temperature and time on the HSSGT flavor profile. The development utilization and improvement of HSSGT requires a comprehensive knowledge of its chemical composition. In this study, the ROAV method was applied to examine the impact of processing on changes to tea aroma, thus providing a more accurate indication of how key aroma compounds in tea alter during different processing stages. This study also employs HS-SPME-GC-MS-O analysis to examine the dynamic changes in chemical properties and aroma volatiles of HSSGT processed under various roasting conditions. The outcomes of this research will provide a theoretical foundation for the mechanized and standardized production of Hubei strip-shaped green tea.

## 2. Materials and Methods

### 2.1. Preparation of Tea Samples

**Figure 1:**
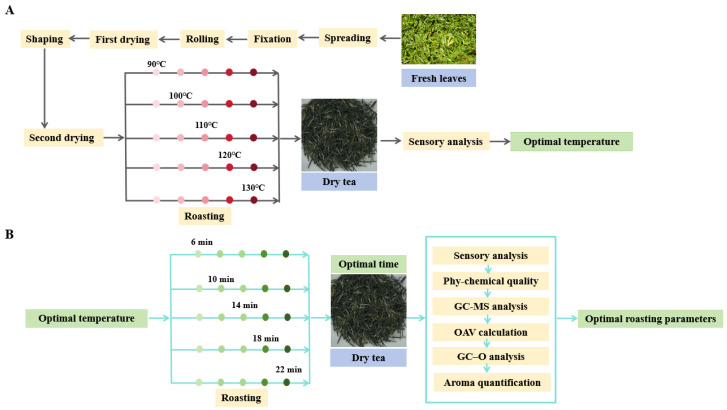
Hubei strip-shaped green tea (HSSGT) manufacturing process. (**A**) Flow chart of HSSGT processing and determination of optimal roasting temperature. (**B**) Flow chart outlining the determination of optimal roasting time and roasting parameter.

On 10 April 2022, in Wufeng County, Hubei, China, ~100 kg of a bud with a leaf from the “E’cha NO 1” tea cultivar was harvested. The fresh leaves measured 3.0–3.5 cm in length. All fresh leaves were processed into secondary drying leaves, which were divided into 10 equal groups, each weighing ~2.7 kg. After roasting, the final weight of each tea sample was ~2.5 kg. The tea samples were processed using traditional methods by an experienced craftsman at Wufeng Jiming Tea Co., Ltd., Hubei, Yichang, China (Figure 1).

(1) Spreading: The harvested leaves were spread for 6–8 h at a thickness of 30–50 mm until their moisture content reached 68–70% (equivalent to 68–70 g of water per 100 g of leaves).

(2) Fixation: The leaves were fixed using a hot roller (model 6CST-80, Zhejiang Shang Yang Co., Ltd., Quzhou, China) for 2.5 min under the following conditions: roller temperatures of 200~240 °C, roller speed of 24 rpm, and fixation time of 72–75 s. The leaves were then cooled and softened in the device (YJY-20, Ningbo Yaojiangyuan Machinery Co., Ltd., Ningbo, China).

(3) Rolling: The fixed leaves were rolled in a rolling machine (6CR-55, Zhejiang Shang Yang Co., Ltd.) for 90 min at a speed of 28 rpm.

(4) First drying: Drying was conducted using a chain plate dryer (model 6CHB-10, Zhejiang Shang Yang Co., Ltd.) at 110 °C until the moisture content was reduced to 24–26%.

(5) Shaping: The partially dried leaves were shaped by a shape-fixing device (model 60K-S, Kawasaki Tea Machine Co., Ltd., Kawasaki, Japan) and dried at 100 °C for 20–22 min, followed by 30 min of cooling outdoors.

(6) Second drying: The shaped leaves were further dried at 100 °C for 15 min using a chain plate dryer (6CHB-10, Zhejiang Shang Yang Co., Ltd.), followed by another 30 min of cooling outdoors.

(7) Roasting: The twice-dried leaves were roasted at varying temperatures (90 °C, 100 °C, 110 °C, 120 °C, and 130 °C) for 10 min using a box-hot air-drying device (model JY-6CHZ-7B, Jiayou Machinery Co., Ltd., Quanzhou, China). The leaves were spread at a thickness of 20–30 mm, with the fan motor power set at 0.55 kW and the motor speed set at 1400 rpm. Sensory quality assessments were conducted to identify the optimal roasting temperature (Figure 1A). Leaves roasted at the optimal temperature were tested at different durations (6 min, 10 min, 14 min, 18 min, and 22 min) to determine the best roasting time (Figure 1B). The 10 samples were prepared in triplicate, with each treatment weighing ~2.5 kg.

### 2.2. Sensory Quality Assessment

A team of five expert tea tasters from the Institute of Fruit and Tea at the Hubei Academy of Agricultural Sciences conducted the sensory evaluations, adhering to the national standards for tea sensory evaluation and terminology standards [13,14]. Each 3 g tea sample was brewed in 150 mL of freshly boiled distilled water for 4 min. The brewed tea was then served in white porcelain bowls, with each sample marked by a random blind code. Every tea sample underwent blind evaluation three times, with each evaluation randomly repeated three times.

The evaluation was completed by well-trained panelists (two males and three females), aged 29–46, who conducted evaluations by smelling and tasting the tea, with a 30 s interval between samples [15]. The panelists were provided with reference samples of different intensities for each characteristic to evaluate and familiarize themselves with the sensations and the corresponding intensity scale. After familiarization, the panelists performed individual assessments. The quality scores of the tea samples were determined using a hundred-mark system, calculated as the average of the scores from the five panelists.

### 2.3. Determination of HSSGT Color Quality

The color quality of HSSGT was assessed using *L*, *a*, and *b* values in a 3D color space. The *L* value measures the brightness (0 = pure black; 100 = pure white). The *a* value ranges from green (negative) to red (positive), and the *b* value reflects the spectrum from blue (negative) to yellow (positive). Dry tea leaves and brewed tea were analyzed for color characteristics using a spectrophotometer (CM-5, Konica Minolta Investment Ltd., Shanghai, China), with distilled water as the baseline measurement control [15,16,17].

### 2.4. Physicochemical Quality Determination

Moisture levels were determined utilizing the 120 °C drying method [18]. Amino acid levels were measured using the ninhydrin colorimetric method [19]. Total tea polyphenols were quantified utilizing the iron tartrate colorimetric method [20], and soluble sugar content was determined by the anthrone colorimetric method [15].

Catechins were analyzed by preparing tea powder using a ball mill (MM400, Retsch, Haan, Germany) with zirconia beads for 1.5 min at 30 Hz. Tea powder (1 g) was mixed with 25 mL of 70% methanol in a 50 mL tube, vortexed for 30 s, and extracted in a 70 °C water bath for 10 min. After centrifugation at 4 °C for 10 min, the resulting supernatant was transferred to a flask. This extraction process was repeated, and the supernatant was filtered through a 0.22 μm microporous membrane for HPLC analysis (Waters 2695, CA, USA) utilizing the external standard method. Catechins were analyzed by HPLC using an Agilent (Santa Clara, CA, USA) ZORBAX SB-Aq C18 column (4.6 mm × 250 mm, 5 μm). The mobile phase included phase A (2% acetic acid in water) and phase B (100% acetonitrile), with an elution gradient of 0–16 min at 6.5–85% B, 16–25 min at 85–75% B, 25–30 min at 75–6.5% B, and 30–40 min at 6.5% B. The column temperature was maintained at 40 °C, the sample injection volume was 10 μL, and the flow rate was 1.0 mL/min [21]. Each sample was performed in triplicate.

### 2.5. Analysis of the Volatile Compounds by Gas Chromatography–Mass Spectrometry (GC-MS)

HSSGT volatile compounds were analyzed using HS-SPME (Supelco, Bellefonte, PA, USA) coupled with GC-MS (Agilent 7890A, 5975C MSD) [16]. A solid-phase micro-extraction (SPME) device was used consisting of a fused silica fiber coated with 50/30 μm divinylbenzene/carboxen/polydimethylsiloxane. The fiber was preconditioned for 20 min in the injection port of the gas chromatograph at 260 °C to remove any volatiles remaining on the fiber before each extraction. For each sample, 3.0 g of dried tea leaves were sealed in a 100 mL vial with 30 mL of boiling water and 20 μL of ethyl caprate (internal standard). The vial was equilibrated in a 50 °C water bath for 10 min, and the SPME fiber was exposed to the headspace for 50 min before desorption at 240 °C for 3 min. Volatile compounds were analyzed using an Agilent 7890A gas chromatograph coupled with a 5975C MSD ion trap mass spectrometer. Separation occurred on a DB-5MS capillary column (30 m × 0.25 mm × 0.32 μm).

The oven temperature was programmed from 50 °C (held for 5 min) to 180 °C at 3 °C/min (held for 2 min), then to 250 °C at 10 °C/min (held for 3 min). Helium, at a flow rate of 1.0 mL/min, was the carrier gas. Mass spectrometric detection used electron impact ionization (70 eV), scanning between 35 and 400 AMU. Compounds were identified utilizing the NIST library and linear retention indices (RIs) based on C7–C40 alkanes as standards [15].

Volatile components were identified by comparing the mass spectra and practical retention indexes (RIs) of these data with those in the NIST 17 database, using a matching score threshold of 70 for the mass spectra. RIs were calculated based on the published retention time of each compound. Relative VC contents were quantified and normalized to ethyl caprate as an internal standard.

### 2.6. ROAV Calculation

Relative odor activity values (ROAVs) were calculated by dividing the concentration of each compound by its odor detection threshold [22]. The volatile components were quantified using the following formula: volatile compounds concentration = (volatile compounds peak area/ethyl caprate peak area) × ethyl caprate concentration, μg/L. Compounds with an ROAV of 1 or greater are considered potential contributors to the tea’s aroma, while those with an ROAV exceeding 10 are regarded as significant contributors to the overall aroma profile [23].

### 2.7. GC–O Analysis

The GC–O analysis was performed similarly to the GC-MS analysis in Section 2.4 with minor modifications. The aroma extract was split evenly between the Olfactory Detection Port (ODP3, Gerstel, Nordrhein-westfalen, Germany) and the MS. The injector and transfer line temperatures were set at 230 °C and 260 °C, respectively, using 99.99% pure nitrogen as the carrier gas.

Three trained assessors from the Fruit and Tea Research Institute of the Hubei Academy of Agricultural Sciences (Section 2.2) conducted the GC-O analysis. Each sample was analyzed twice, and aroma intensities (AI) were scored on a 4-point scale, where “1” = weak, “2” = moderate, “3” = strong, and “4” = extremely strong [24].

### 2.8. Data Analysis

Mean values from three independent measurements were analyzed utilizing one-way ANOVA and Duncan’s multiple-range tests using SPSS 23.0 (Demo version, Armonk, NY, USA). Figures were created using GraphPad Prism v8.0.2.263 (GraphPad Software Inc., San Diego, CA, USA).

## 3. Results

### 3.1. Effect of Varying Roasting Treatments on HSSGT Sensory Qualities

**Figure 2:**
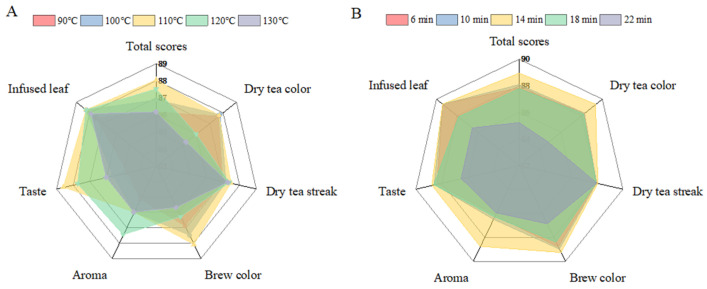
Sensory quality scores of roasted HSSGT. (**A**) Sensory scores at different temperatures. (**B**) Sensory scores for varying roasting times at 110 °C.

The sensory assessment of HSSGT under different roasting treatments is presented in Figure 2A. The analysis indicated that samples roasted at 110 °C achieved the highest sensory scores, meeting the high-quality standards for HSSGT. These improvements are likely due to the oxidation and degradation of phenolic compounds or the formation of Maillard reaction products [16,25,26,27]. Consistent with prior studies, drying temperature significantly affects green tea’s umami taste, bitterness, and astringency. Thus, 110 °C was identified as the optimal roasting temperature for HSSGT processing.

A comprehensive evaluation of HSSGT roasted at 110 °C for various durations is presented in Figure 2B. Tea roasted for 14 min received the highest score, followed by tea roasted for 10, 6, 18, and 22 min. Roasting duration notably impacted the color of dry tea, liquor color, aroma, and taste but had minimal effect on the appearance of dry tea streaks and infused leaves. Tea roasted for 14 min exhibited a tightly curled, heavy appearance, bright green color, a chestnut and floral aroma, and a mellow, rich flavor, with significantly higher scores for the color of dry tea, aroma, and taste (*p* < 0.05). Similarly, tea roasted for 6 and 10 min also had a tightly curled, heavy appearance with green color, chestnut, and slightly green aromas, with a mellow and rich flavor. In contrast, tea roasted for 18 and 22 min displayed a green-yellow color, chestnut, and strong roasted aromas with a thicker but slightly bitter taste. These results align with previous research on yellow tea [15], emphasizing the critical role of roasting time in producing stable, high-quality tea with sweet and floral aromas.

### 3.2. Impact of Varying Roasting Time on HSSGT Color Quality

**Figure 3:**
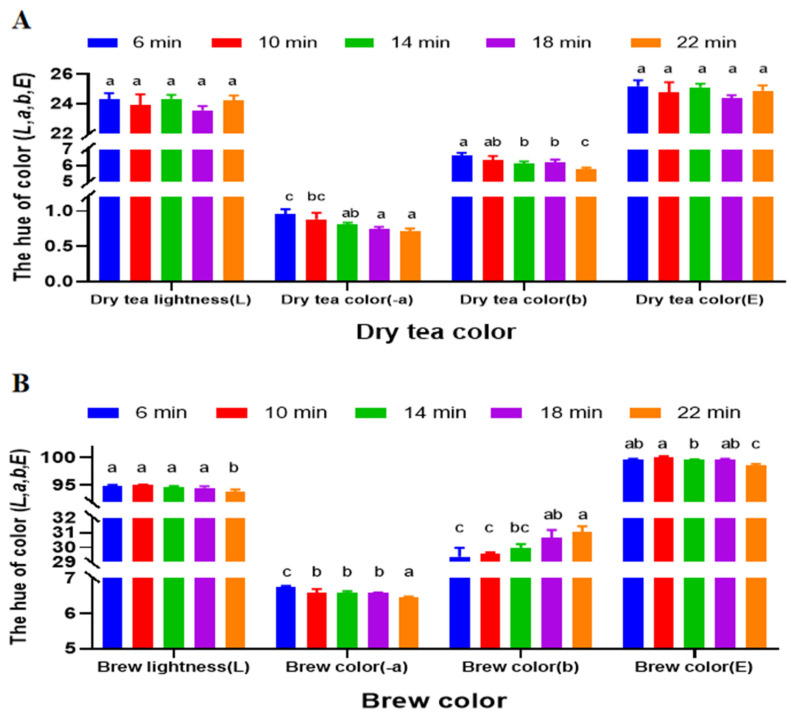
Color quality scores of HSSGT roasted at 110 °C for different durations. (**A**) Dry tea color analysis. (**B**) Brew color analysis. Each sample was performed in triplicate. Columns labeled with ‘a’, ‘b’, and ‘c’ had significant differences (*p* < 0.05) from each other.

The effect of varying roasting durations on the color quality of dried and brewed HSSGT is illustrated in Figure 3. Roasting time significantly influenced hue components, particularly the *a* and *b* values. As roasting time increased, dried HSSGT became progressively more yellow. No significant lightness (*L* value) and color (*E* value) differences, however, were observed among the treatments. Additionally, longer roasting durations resulted in darker and redder tea infusions, with increased *a* and *b* values. These findings are consistent with previous studies on oolong teas, which demonstrate that dry-heating enhances tea taste and aroma, improving both color and flavor [28]. The reason for this phenomenon is that during the roasting process, chemical reactions were underway, such as enzymatic browning, pigment oxidation, Maillard reactions [29,30], polyphenol oxidation, isomerization, starch hydrolysis, and protein decomposition, leading to partial damage and in chemical composition changes [31,32].

### 3.3. Impact of Roasting Time on Bioactive Compound Content

**Figure 4:**
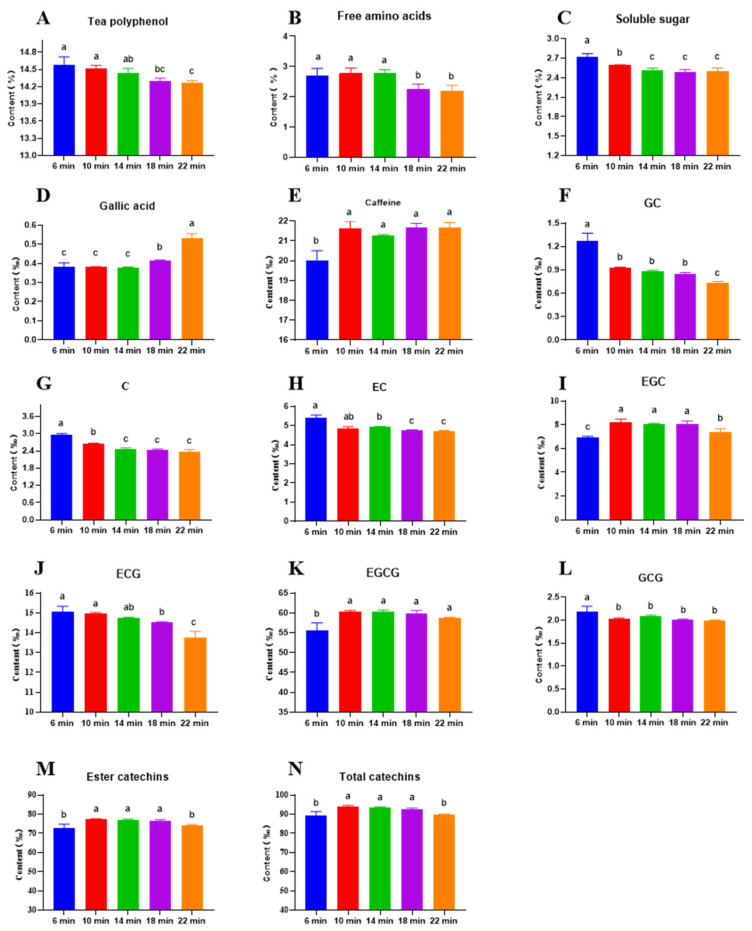
Changes in bioactive compound content of HSSGT roasted at 110 °C for varying durations. (**A**) Tea polyphenol; (**B**) Free amino acids; (**C**) Soluble sugar; (**D**) Gallic acid; (**E**) Caffeine; (**F**) GC; (**G**) C; (**H**) EC; (**I**) EGC; (**J**) ECG; (**K**) EGCG; (**L**) GCG; (**M**) Ester catechins; (**N**) Total catechins. Columns labeled with ‘a’, ‘b’, and ‘c’ had significant differences (*p* < 0.05) from each other.

The impact of roasting time on the chemical composition of HSSGT is depicted in Figure 4. Key components, including tea polyphenols (Figure 4A), free amino acids (Figure 4B), soluble sugars (Figure 4C), gallic acid, caffeine, and seven catechins, such as the C (catechin), EC (epicatechin), GC (gallo catechin), ECG (epicatechin gallate), EGC (epigallocatechin), GCG (gallocatechin gallate), and EGCG (epigallocatechin gallate), were identified and quantified in the tea samples. Longer roasting time significantly decreased the levels of polyphenols, soluble sugars, and free amino acids, as well as GC, C, EC, ECG, and GCG, while increasing gallic acid, caffeine, and EGCG levels. Interestingly, the EGC, ester catechins, and total catechins initially increased and then decreased over time. These findings highlight the substantial influence of roasting time on the physicochemical composition of HSSGT, which in turn influences its taste, sensory qualities, and color.

Polyphenols (Figure 4A), known for imparting bitterness and astringency [33,34], and amino acids and sugars, which provide umami and sweetness [35], were significantly higher in the early roasting stages (6–14 min), but gradually decreased during later stages (14–22 min). Interestingly, tea roasted for 14 min exhibited the highest concentrations of these key flavor compounds, which optimized the formation of desirable flavors. Gallic acid (Figure 4D), which imparts astringency [36], remained relatively unchanged in the early roasting stages (6–14 min) but increased slowly in the later stages (14–22 min). Caffeine (Figure 4E) content increased during the early roasting stages (6–14 min) and then stabilized during the later stages (14–22 min). The variations in total polyphenols, caffeine, and gallic acid were related to non-enzymatic reactions, including polymerization, hydrolysis, and Maillard reaction, while the longer roasting time could enhance those reactions, which led to the higher content of total polyphenols in the 6–10 min and then gradually decreased in the 14–22 min. Caffeine and gallic acid content were higher in the 18–22 min. These findings, combined with the sensory quality results, provide valuable insights for optimizing HSSGT processing relative to roasting time.

Seven catechins (Figure 4F–L) HSSGT underwent dynamic changes under different roasting treatments. GC, C, EC, ECG, and GCG were notably higher during the early roasting stage (6–14 min), while EGC levels increased in the early stage (6–10 min), remained stable in the middle stage (10–18 min), and slowly decreased in the late stage (18–22 min). EGCG increased in the early stage (6–10 min) and then stabilized during the middle and late roasting stages (10–22 min). Ester catechins, such as ECG, GCG, and EGCG, are among the most biologically active compounds in tea and affect the overall flavor profile [34]. In this study, ester catechins (Figure 4M) and total catechins (Figure 4N) increased during the early roasting stage (6–10 min), remained stable in the middle stage (10–18 min), and gradually decreased during the late stage (18–22 min). These findings suggest that HSSGT roasted for 10–18 min contained higher levels of health-beneficial catechins but also imparted a more astringent and bitter characteristic to the tea infusion.

HSSGT roasted for 10 min contained a relatively low content of gallic acid, caffeine, EGC, EGCG, ester catechins, and total catechins, whereas tea roasted for 18 min had the lowest amount of tea polyphenols, soluble sugars, free amino acids, GC, C, EC, ECG, GCG, ester catechins, and total catechins levels. We speculated that interactions between the amino acids and soluble sugars (umami and sweetness) and different catechins (GC, C, EC, ECG, GCG, and EGCG) impacted tea flavor and aroma; these compounds are involved in the Maillard reaction and impact tea flavor [37]. Longer roasting at high temperatures increased GCG and GA content, possibly due to ester catechin degradation [38,39,40]. At the medium roasting stage (10–14 min), polyphenols, ECG, and GCG may have transformed into EGC and other non-ester catechins. The consistent presence of GC, EGC, and EGCG in all HSSGT samples suggests that these catechins, particularly EGCG, are major contributors to tea flavor. Overall, the findings suggest a roasting duration between 10 and 14 min optimized HSSGT taste and color characteristics.

### 3.4. Profile of HSSGT Volatile Compounds After Roasting for Different Durations

**Figure 5:**
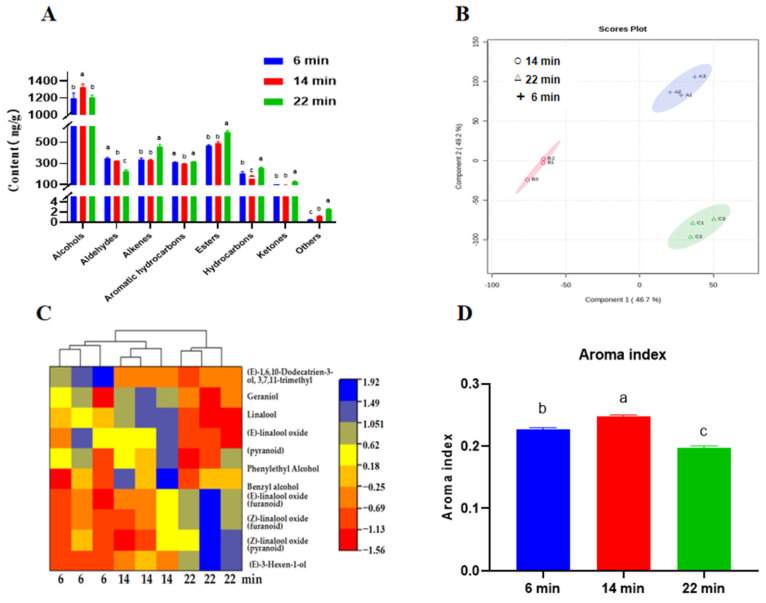
Characterization of HSSGT VOCs. (**A**) The abundance of specific categories of VOCs in HSSGT roasted for 6, 14, and 22 min. (**B**) Principal component analysis. (**C**) Heat map of main differential alcohol compounds. (**D**) Aroma index. Columns labeled with ‘a’, ‘b’, and ‘c’ had significant differences (*p* < 0.05) from each other.

Based on sensory and chemical analyses, samples roasted for 6, 14, and 22 min were selected to evaluate the volatile compound profile using HS-SPME-GC-MS. It is noteworthy that consumers prefer HSSGT with chestnut-like and flowery aromas. Seventy volatile organic compounds (VOCs) were identified (Figure 5A) and classified into eight categories: alcohols, aldehydes, alkenes, aromatic hydrocarbons, esters, hydrocarbons, ketones, and others. Among these, alcohols were the most prevalent in all samples, contributing 36.67% (6 min), 41.16% (14 min), and 34.95% (22 min) of the total VOCs. This finding aligns with previous studies [41,42].

We used a PLS-DA model to further analyze the characteristic aroma components (Figure 5B), which yielded fitting indices of R^2^X = 0.968, R^2^Y = 0.934, and Q^2^ = 0.928. From the PLS-DA, six volatile substances were identified with variable importance in projection (VIP) scores exceeding 1, with linalool scoring the highest (VIP > 3). Linalool imparts floral, fruity, and woody aromas [9]. Alcohol compounds are critical aroma contributors in green tea; for instance, geraniol imparts a fruity, floral, and sweet aroma [43], and benzyl alcohol adds a sweet and citrus scent [42,44]. The predominant alcohols identified in HSSGT roasted for 6 min were linalool, geraniol, (Z)-linalool oxide (pyranoid), and benzyl alcohol, contributing 15.78%, 5.09%, 3.29%, and 3.64% of total VOCs, respectively. With a roasting time of 14 min, the levels of these alcohols rose, reaching 17.75%, 5.33%, 3.34%, and 4.35%, respectively. However, with a roasting time of 22 min, the levels of these alcohols decreased, except for (Z)-linalool oxide (pyranoid) (Figure 5C). Consequently, the aroma index of HSSGT roasted for 14 min was the highest (*p* < 0.05) (Figure 5D). Since alcohols are key odorants responsible for chestnut and floral aromas [9], the results suggest that linalool, geraniol, (Z)-linalool oxide (pyranoid), and benzyl alcohol play critical roles in imparting the fresh and chestnut and floral aroma characteristics of HSSGT.

Aldehyde compounds, commonly present in green tea, also contribute significantly to its aroma profile. Notable aldehydes include nonanal, which imparts a rose fragrance; benzaldehyde, with an almond odor [45]; citral, with a lemony taste; phenylacetaldehyde, with a honey scent [9]; and trans-2,4-heptadienal, which gives a grassy, fresh aroma [46]. In this study, we detected aldehydes in the HSSGT samples (Figure 5A), such as benzaldehyde and phenylacetaldehyde. These components decreased as roasting time increased (Table 1). Therefore, we speculated that prolonged high temperatures led to the degradation of some components into alkanes. Among the 10 detected alkenes, (*E*)-4,8-dimethylnona-1,3,7-triene has been identified as a key chestnut-like odorant in green tea [47,48]. In addition, (*E*,*E*)-3,5-octadien-2-one imparts a key chestnut-like odorant in Longjing green tea [24], and β-ocimene provides a warm, floral, and herbal fragrance [42]. Interestingly, the 22 min roasting treatment had the highest alkene content (*p* < 0.05). Notably, the levels of β-ocimene in the 22 min sample (Table 1) were twice as high as in the 14 min roasted sample (*p* < 0.01). These compounds are considered primary contributors to tea’s floral, fruity, and delicate aroma. Their combined effect contributed to the formation of both chestnut-like and floral aromas in HSSGT roasted for 14 min.

HSSGT also contained six hydrocarbons, nine esters, and three ketones. The role of hydrocarbons in tea flavor is debated, with some studies suggesting minimal impact [49]. However, recent research has found that certain hydrocarbons, such as o-cymene, contribute to aromatic qualities [39], while naphthalene, 2-methyl-naphthalene, and 1,2-dihydro-1,1,6-trimethyl-naphthalene are critical contributors to clean and chestnut-like aromas [27]. In this study, the content of o-Cymene in the 22 min roasted sample was notably higher compared to other roasting durations (Table 1), whereas the concentrations of naphthalene, 2-methyl-naphthalene, and 1,2-dihydro-1,1,6-trimethyl-naphthalene decreased with longer roasting times. These results indicated that the 14 min roasting treatment had the highest content of compounds contributing to a clean and chestnut-like aroma.

Esters, such as methyl salicylate (mint aroma) [50], *cis*-3-hexenyl butyrate (floral, fruity and delicate) [51], (Z)-3-Hexenyl hexanoate (floral, fruity, sweet) [42], *cis*-3-hexenyl *cis*-3-hexenoate, dihydroactinidiolide (chestnut-like), acetic acid lactone (green leaf and citrus fragrance) [9], coumarin (sweet and bitter) [52] have minimal impact on HSSGT flavor [53]. Although the ketone content was relatively low (Figure 5A), ketones are known to significantly influence tea aroma due to their low aroma threshold values and high flavor dilution (FD) factors [54]. The most abundant ketones in HSSGT were (Z)-jasmone (floral, herbal, spicy) [42], sulcatone (citrus, green, lemongrass, apple) [28], and β-ionone (violet-like, raspberry, floral) [40]. Interestingly, the content of these key esters and ketones increased in the 22 min roasted HSSGT (*p* < 0.05), resulting in a chestnut-like aroma with a hint of caramel.

### 3.5. Comparison of the Relative Odor Activity Values of Key HSSGT Odorants After Roasting

The roasting process of strip green tea involves a series of complex chemical changes, causing fluctuating aroma compound levels over time [55]. Analyzing the changes in ROAVs of key compounds in HSSGT is essential to understanding how these aroma components develop during roasting.

In this study, we compared ROAVs of 16 key HSSGT aroma compounds roasted for 6, 14, and 22 min (Table 1). Among the 16 key odorants, β-ionone (violet, woody) contributed the most to the chestnut-like and floral aroma due to its high ROAV(1676–1809) compared to other compounds, followed by β-ocimene (warm, floral, herbal, sweet, ROAV = 1286–2348), *trans*-calamanene (herbal, spicy, ROAV = 748–1163). These results indicated that β-ionone, β-ocimene, and *trans*-calamenene are dominant contributors to the chestnut-like aroma quality of HSSGT. The odorants with ROAVs > 10 across different roasting durations included heptanal, octanal, β-ocimene, linalool, nonanal, geraniol, 3-hexenyl hexanoate, jasmone, β-ionone, δ-cadinene, and *trans*-calamanene. β-ocimene, jasmone, and *trans*-calamanene increased with roasting time (*p* < 0.05), while β-ionone levels showed no significant change (*p* > 0.05). The level of linalool was the only odorant that significantly increased in the 14 min roasting treatment, whereas other odorants, such as heptanal, octanal, nonanal, geraniol, and δ-cadinene decreased (*p* < 0.05).

While the other compounds VOCs with ROAVs between 1 and 10 included *trans*, *trans*-2,4-heptadienal (green, grassy, fresh), hexanal (strong green, grassy, fruity), o-cymene (aromatic), benzeneacetaldehyde (sweet, floral honey, rosy), and *cis*-3-hexenyl *cis*-3-hexenoate (tender, fresh, clean). Except for o-cymene, the remaining compounds decreased with roasting time. These compounds contributed to the floral attribute of HSSGT but had a minor impact on the overall aroma. The findings indicate that roasting for 14 min is optimal for producing a chestnut fragrance with a floral undertone in HSSGT.

### 3.6. GC-O Analysis of Aroma Compounds in HSSGT Roasted with Different Times

GC-O analysis, which combines GC-MS with human olfactometry, is widely used for identifying key aroma compounds and is extensively used in food flavor analysis [24]. The volatile compounds detected through GC-O are the most important contributors to tea aroma. In this study, GC-O analysis identified 14 key aroma compounds in HSSGT, with average aroma intensities (AI) ranging from 0 to 2.7 (Table 2). Linalool exhibited the highest average aroma intensity (AI = 2.7), and its intensity increased with increased roasting time (*p* > 0.05). Linalool, known for its strong, sweet, and floral aroma, was identified as the primary aroma compound in HSSGT. Other significant aroma compounds included nonanal (sweet aroma, AI = 2.6), 1-octen-3-ol (sweet and floral aroma, AI = 2.0), octanal (green aroma, AI = 1.6), and heptaldehyde (green aroma, AI = 1.4). Previous research has indicated that linalool, nonanal, and octanal are the most prominent odorants responsible for the chestnut-like aroma in green tea [56]. Nonanal, distinguished by its strong odor intensity, significantly influences the sweet floral flavor profiles of black tea and steamed green tea [55]. 1-octen-3-ol, which has a fungal floral aroma, is abundant in Fu brick tea and contributes to the clean aroma of green tea [27,57]. Octanal was highlighted by GC-O (Table 2), consistent with previous research [57,58]. Overall, these results indicated that linalool, nonanal, and 1-octen-3-ol contribute the most to the floral aroma profile of HSSGT. Additionally, compounds such as dimethyl sulfide, 2,2,6-trimethylcyclohexanone, *cis*-linalool oxide, and (E)-3-hexenyl butanoate exhibited chestnut-like, baked, caramel, and roasted-like aromas, suggesting they are likely responsible for the chestnut-like aroma of HSSGT. The synergetic interactions among different odorants may also indirectly enhance the chestnut-like and floral aromas in HSSGT. However, variations in odor descriptions may occur due to differing compound concentrations. To confirm the specific contribution of these compounds to the floral aroma of HSSGT, further absolute quantitative experiments are needed.

### 3.7. Absolute Quantification of Key Aroma Compounds in the HSSGT

As previously discussed, the 16 odorants with ROAVs > 1 in HSSGT were heptanal, octanal, β-ocimene, linalool, nonanal, geraniol, 3-hexenyl hexanoate, jasmone, β-ionone, δ-cadinene, trans-calamanene, *trans*, *trans*-2,4-heptadienal, hexanal, o-cymene, benzeneacetaldehyde, and *cis*-3-hexenyl *cis*-3-hexenoate. Additionally, the 11 odorants with aroma intensities (AI) > 1 identified by GC-O were linalool, nonanal, 1-octen-3-ol, octanal, dimethyl sulfide, heptaldehyde, *cis*-linalool oxide, β-cyclocitral, (-)-α-cubebene, hexanal, and 2,2,6-trimethylcyclohexanone. A combined GC-O and ROAV analysis revealed that linalool, nonanal, octanal, and hexanal were consistently identified as key volatile compounds and, therefore, likely to contribute most to HSSGT chestnut-like and floral aromas. However, discrepancies between the GC-O and ROAV results were observed, likely due to differences in sample preparation methods and subjective factors involved in the analyses.

**Figure 6:**
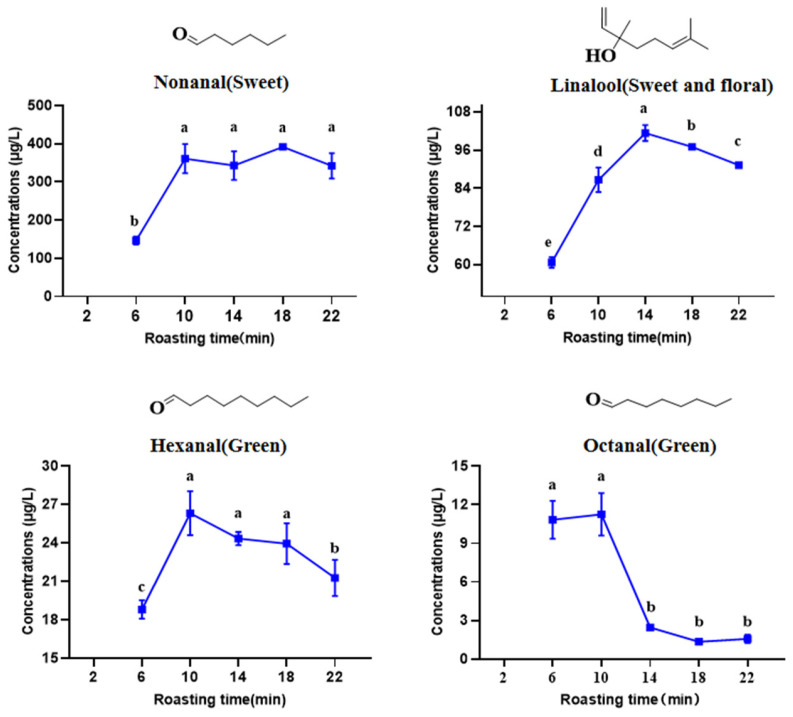
Changes in the abundance of nonanal, linalool, hexanal, and octanal in the HSSGT roasting process. The different letters (‘a’, ‘b’, and ‘c’) indicate significant differences (*p* < 0.05).

To further confirm that linalool, nonanal, octanal, and hexanal are the predominant aroma compounds, absolute quantification using the external standard method was conducted to measure their contents in HSSGT. The results (Table 3) indicated the concentrations of these four compounds as follows: linalool (58.99–106.30 μg/L), nonanal (135.86–380.82 μg/L), octanal (1.3–9.4 μg/L), and hexanal (19.53–28.04 μg/L), which indicated that these absolute quantitative results differed significantly from the relative quantification results from the ROAV analysis (Table 2). For example, the levels of linalool, nonanal, octanal, and hexanal in the 14 min roasting treatment were 12-fold, 15-fold, 1.5-fold, and 1.5-fold higher, respectively, when measured by absolute quantification (Figure 6). These findings suggest that the polarity differences between aroma components and the water solution were minimized when using internal standards, thus leading to more accurate concentration measurements. Additionally, certain components may form hydrogen bonds with water molecules, resulting in lower values when using relative quantification methods. Therefore, absolute quantitative analysis is essential for accurately investigating aroma compounds in food research.

### 3.8. The Possible Formation Pathways of Key Odorants in HSSGT During the Roasting Process

**Figure 7:**
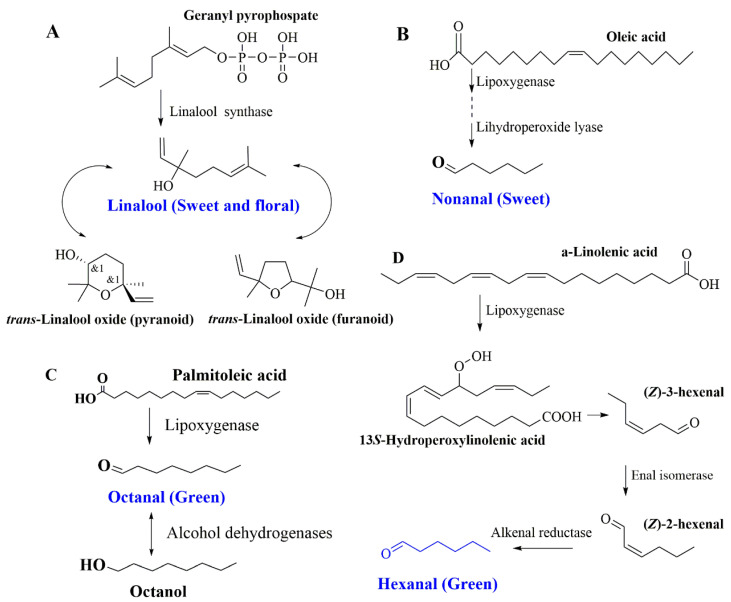
Proposed pathways for molecular formation of the four characteristic HSSGT aroma compounds: (**A**) linalool, (**B**) nonanal, (**C**) octanal, (**D**) hexanal.

We examined the potential mechanisms behind the development of enhanced sweet and floral aromas and the reduction in green odorants in HSSGT during roasting. Previous studies identified linalool as a critical green tea aroma compound, contributing to floral and clean aroma [59], while hexanal and octanal are responsible for green odors, originating from the degradation of α-linolenic acid and linoleic acid [44]. Our findings revealed that medium roasting effectively reduces green odors while enhancing sweet and floral aromas in HSSGT, which resulted in a significant decrease in octanal and hexanal content after 14 min roasting. We propose a molecular mechanism for the formation of these four characteristic HSSGT aroma compounds (Figure 7). Linalool, a key green tea odorant imparting sweet and floral notes, is derived from geranyl pyrophosphate through the action of linalool synthase [44,60]. Nonanal, contributing a sweet aroma, is produced from oleic acid via enzymatic oxidation [59]. Octanal and hexanal, responsible for green odors, are derived from palmitoleic acid and α-linolenic acid, respectively. We propose that the exterior-to-interior heat transfer during the HSSGT roasting process promotes protein degradation and other chemical reactions, resulting in increased nonanal, linalool, and hexanal content in the early to middle stages of the roasting process (*p* < 0.05), followed by a decline in linalool, hexanal, and octanal in the later stages. As a result, the 14 min roasting treatment produces a more pronounced chestnut-like and floral aroma.

## 4. Conclusions

HSSGT, a trendy and economically significant beverage in China, is renowned for its unique chestnut-like aroma and floral flavor. Despite its significance, the impact of roasting on the chemical properties of strip green tea remains inadequately understood. This study used a systematic approach to identify the optimal roasting conditions for HSSGT. Sensory-guided, ROAV, GC-O, and absolute quantification analyses were conducted to identify the predominant aroma compounds responsible for the chestnut-like and floral characteristics of HSSGT. The results indicated that the 14 min roasting treatment had the highest aroma index (*p* < 0.05), and β-ionone, β-ocimene, and *trans*-calamenene were identified as the dominant compounds contributing to the chestnut-like aroma, while hexanal, o-cymene, benzeneacetaldehyde, *cis*-3-hexenyl, and *cis*-3-hexenoate were key components of the floral aroma. The optimized roasting condition of 110 °C for 14 min enhanced the proportion of chestnut and floral volatile compounds and their ROVAs. Furthermore, linalool, nonanal, octanal, and hexanal were identified for the first time as the characteristic aroma compounds of HSSGT. These results highlight the importance of roasting conditions in enhancing the quality of strip green tea, emphasizing their role in achieving the desirable chestnut and floral aroma in HSSGT processing.

## Figures and Tables

**Table 1:** Odor descriptions and relative odor activity values (ROAVs) of volatile compounds in HSSGT roasted for 6, 14, and 22 min.

No.	Volatile Compounds *	Rt (min)	CAS	RI/NIST RI	Odor Descriptions	Threshold (μg/kg) *	ROVA (Mean ± SD)		
							6 min	14 min	22 min
1	β-Ionone	31.17	79-77-6	1478	Violet-like, raspberry, floral	0.007	1695 ± 92 ^a^	1809 ± 67 ^a^	1676 ± 166 ^a^
2	β-Ocimene	18.60	1040397-46-3	1045	Warm, floral, herbal, sweet	0.02	1286 ± 131 ^b^	1576 ± 137 ^b^	2348 ± 286 ^a^
3	*trans*-Calamenene	32.34	73209-42-4	1525	Herbal, spicy	0.02	748 ± 99 ^b^	751 ± 50 ^b^	1163 ± 59 ^a^
4	δ-cadinene	31.85	13862-36-7	1520	Herbal, woody	1.5	87 ± 2 ^a^	63 ± 3 ^b^	58 ± 1 ^c^
5	Jasmone	28.92	488-10-8	1391	Floral, creamy	1.9	26.7 ± 3.3 ^b^	28.1 ± 1.3 ^b^	45.7 ± 1.4 ^a^
6	Geraniol	24.835	103606-17-3	1246	Rose-like	7.5	21.6 ± 1.6 ^ab^	23.0 ± 0.3 ^a^	20.6 ± 0.5 ^b^
7	(Z)-3-Hexenyl hexanoate	28.43	294177-31-4	1373	Floral, fruity, sweet	16	18.1 ± 0.5 ^b^	18.7 ± 0.3 ^b^	22.3 ± 0.5 ^a^
8	Nonanal	22.47	124-19-6	1102	Fat, citrus, green	1	16.4 ± 3.2 ^b^	23.1 ± 0.7 ^a^	17.0 ± 1.1 ^b^
9	Heptanal	13.73	111-71-7	900	Chestnut-like, sweet, grass	3	10.9 ± 0.2 ^a^	9.9 ± 0.3 ^a^	5.1 ± 0.9 ^b^
10	Octanal	17.16	124-13-0	1001	Fat, soap, lemon, green	0.7	9.2 ± 0.5 ^a^	6.8 ± 0.2 ^b^	4.9 ± 0.2 ^c^
11	Hexanal	10.4	66-25-1	799	Strong green, grassy, fruity	4.5	6.1 ± 0.2 ^a^	5.9 ± 0.6 ^b^	4.8 ± 0.4 ^c^
12	Benzeneacetaldehyde	18.56	122-78-1	1044	Sweet, floral honey, rosy	4	2.8 ± 0.1 ^a^	3.0 ± 0.9 ^a^	1.3 ± 0.1 ^b^
13	*trans*, *trans*-2,4-Heptadienal	17.43	5910-85-0	1009	Fatty, green odor	15.4	1.9 ± 0.1 ^a^	1.8 ± 0.1 ^ab^	1.6 ± 0.1 ^b^
14	*cis*-3-Hexenyl *cis*-3-hexenoate	28.53	61444-38-0	1377	Tender, fresh, clean	16	1.2 ± 0.2 ^b^	1.2 ± 0.0 ^b^	1.6 ± 0.1 ^a^
15	Linalool	20.32	78-70-6	1098	Pleasant floral	6	1.0 ± 0.1 ^b^	1.3 ± 0.0 ^a^	0.97 ± 0.0 ^c^
16	o-Cymene	17.97	527-84-4	1026	Aromatic	11.4	1.0 ± 0.1 ^b^	1.82 ± 0.0 ^a^	1.67 ± 0.1 ^a^

* The thresholds of compounds in water refer to Reference [27]. Rt: retention time. ROVA: relative odor activity value. Columns labeled with ‘a’, ‘b’, and ‘c’ had significant differences (*p* < 0.05) from each other.

**Table 2:** GC-O identification in HSSGT roasted for 6, 14, and 22 min.

No.	Rt (min)	Compounds	Odor Descriptions	Average AI	AI		
					6 min	14 min	22 min
1	2.06	Dimethyl sulfide	Sweet, chestnut-like	1.5	1.5 ± 0.2	1.5 ± 0.2	1.5 ± 0.2
2	4.73	Hexanal	Green	1.2	1.5 ± 0.3	1.0 ± 0.2	1.0 ± 0.2
3	8.07	Heptaldehyde	Green	1.4	1.5 ± 0.2	1.8 ± 0.2	1.8 ± 0.3
4	11.79	1-Octen-3-ol	Sweet and floral	2.0	2.5 ± 0.3	2.0 ± 0.3	2.0 ± 0.3
5	13.20	Octanal	Green	1.6	1.5 ± 0.2	1.9 ± 0.3	1.5 ± 0.2
6	14.43	2,2,6-Trimethylcyclohexanone	Like burnt, baked, pungent	1.0	1.0 ± 0.2	1.0 ± 0.2	1.0 ± 0.2
7	17.83	*cis*-Linalool oxide	Earthy floral, caramel-like	1.2	1.5 ± 0.3	1.0 ± 0.2	1.0 ± 0.2
8	18.02	Linalool	Sweet and floral	2.7	2.5 ± 0.3	2.8 ± 0.3	3.0 ± 0.3
9	18.18	Nonanal	Sweet	2.6	2.0 ± 0.3	2.8 ± 0.3	3.0 ± 0.3
10	22.15	(E)-3-hexenyl butanoate	Baked, pungent	0.8	1.5 ± 0.2	0.8 ± 0.2	-
11	23.13	β-cyclocitral	Green, fruity	1.2	1.5 ± 0.2	1.0 ± 0.2	1.0 ± 0.2
12	28.89	(-)-α-Cubebene	Tender floral	1.2	1.5 ± 0.2	1.0 ± 0.2	1.0 ± 0.2
13	30.88	(Z)-hex-3-en-1-yl hexanoate	Tender floral, fresh	1.0	1.0 ± 0.2	1.0 ± 0.2	1.0 ± 0.2
14	39.81	Cedrol	Tender floral	1.0	1.0 ± 0.2	1.0 ± 0.2	1.0 ± 0.2

Data are means ± SD. Rt: retention time. AI means aroma intensities.

**Table 3:** Standard curves of key aroma-active compounds of HSSGT.

No.	Compounds	Standard Curve	R^2^	Linear Range (μg/L)
1	Linalool	Y = 0.79 × 10^−6^X − 2.08	0.9841	2.07~20,654.90
2	Nonanal	Y = 0.23 × 10^−3^X − 315.75	0.9924	1.79~17,866.70
3	Octanal	Y = 4.74 × 10^−8^X − 0.033	0.9983	0.82~82.00
4	Hexanal	Y = 6.41 × 10^−6^X + 1.1548	0.9973	1.31~13,102.80

## Data Availability

All the data generated during this study are provided in the manuscript.

## References

[1] Liu Z., Gao L., Chen Z., Zeng X., Huang J.A., Gong Y., Li Q., Liu S., Lin Y., Cai S. (2019). Leading progress on genomics, health benefits and utilization of tea resources in China. Nature.

[2] Zhang Y., Yan K., Peng Q., Zhu Y., Dai W., Fu J., Lv H., Lin Z., Shi J., Baldermann S. (2024). Comprehensive analysis of pigment alterations and associated flavor development in strip and needle green teas. Food Res. Int..

[3] Li Y., Ran W., He C., Zhou J., Chen Y., Yu Z., Ni D. (2022). Effects of different tea tree varieties on the color, aroma, and taste of Chinese Enshi green tea. Food Chem. X.

[4] Zou D., Yin X.-L., Gu H.-W., Peng Z.-X., Ding B., Li Z., Hu X.-C., Long W., Fu H., She Y. (2024). Insight into the effect of cultivar and altitude on the identification of EnshiYulu tea grade in untargeted metabolomics analysis. Food Chem..

[5] Fei Y., Ziming G., Anhui G., Jing T., Shiwei G. (2019). Physico-chemical characteristics and quality improvement of machine picking green tea by automatic production line. Trans. Chin. Soc. Agric. Eng. Trans. CSAE.

[6] Ouyang W., Yu Y., Wang H., Jiang Y., Hua J., Ning J., Yuan H. (2022). Analysis of volatile metabolite variations in strip green tea during processing and effect of rubbing degree using untargeted and targeted metabolomics. Food Res. Int..

[7] Jeszka-Skowron M., Frankowski R., Zgoła-Grześkowiak A. (2020). Comparison of methylxantines, trigonelline, nicotinic acid and nicotinamide contents in brews of green and processed Arabica and Robusta coffee beans—Influence of steaming, decaffeination and roasting processes on coffee beans. LWT Food Sci. Technol..

[8] Yang Y., Qian M.C., Deng Y., Yuan H., Jiang Y. (2022). Insight into aroma dynamic changes during the whole manufacturing process of chestnut-like aroma green tea by combining GC-E-Nose, GC-IMS, and GC × GC-TOFMS. Food Chem..

[9] Zhu Y., Lv H.P., Shao C.Y., Kang S., Zhang Y., Guo L., Dai W.D., Tan J.F., Peng Q.H., Lin Z. (2018). Identification of key odorants responsible for chestnut-like aroma quality of green teas. Food Res. Int..

[10] Jiang Z., Han Z., Wen M., Ho C.T., Wu Y., Wang Y., Xu N., Xie Z., Zhang J., Zhang L. (2022). Comprehensive comparison on the chemical metabolites and taste evaluation of tea after roasting using untargeted and pseudotargeted metabolomics. Food Sci. Hum. Wellness.

[11] Fu Y.Q., Wang J.Q., Chen J.X., Wang F., Xu Y.Q. (2020). Effect of baking on the flavor stability of green tea beverages. Food Chem..

[12] Weng Y., Chen L., Kun J., He S., Tong H., Chen Y. (2025). The unique aroma of ripened Pu-erh tea, Liupao tea and Tietban tea: Associated post-fermentation condition and dominant microorganism with key aroma-active compound. Food Chem..

[13] (2018). Methodology for Sensory Evaluation of Tea.

[14] (2017). Tea Vocabulary for Sensory Evaluation.

[15] Ye F., Qiao X., Gui A., Liu P., Wang S., Wang X., Teng J., Zheng L., Feng L., Han H. (2022). Characterization of Roasting Time on Sensory Quality, Color, Taste, and Nonvolatile Compounds of Yuan An Yellow Tea. Molecules.

[16] Cao Q.Q., Wang F., Wang J.Q., Chen J.X., Yin J.F., Li L., Meng F.K., Cheng Y., Xu Y.Q. (2021). Effects of brewing water on the sensory attributes and physicochemical properties of tea infusions. Food Chem..

[17] Ye F., Qiao X., Gui A., Wang S., Liu P., Wang X., Teng J., Zheng L., Feng L., Han H. (2021). Metabolomics Provides a Novel Interpretation of the Changes in Main Compounds during Black Tea Processing through Different Drying Methods. Molecules.

[18] (2017). Tea-Determination of Moisture Content.

[19] (2003). Determination of Amino Acids in Foods.

[20] (2005). Determination of Substances Characteristic of Green and Black Tea—Part 1: Content of Total Polyphenols in Tea—Colorimetric Method Using Folin-Ciocalteu Reagent.

[21] Wang H., Hua J., Jiang Y., Yang Y., Yuan H. (2020). Influence of fixation methods on the chestnut-like aroma of green tea and dynamics of key aroma substances. Food Res. Int..

[22] Liu P., Zheng P., Feng L., Gong Z., Zheng L., Gao S., Wang X., Ye F., Huang J., Liu Z. (2022). Dynamic changes in the aroma profile of Qingzhuan tea during its manufacture. Food Chem..

[23] Schieberle P. (1995). New Developments in Methods for Analysis of Volatile Flavor Compounds and their Precursors. Characterization of Food.

[24] Wang M.Q., Ma W.J., Shi J., Zhu Y., Lin Z., Lv H.P. (2020). Characterization of the key aroma compounds in Longjing tea using stir bar sorptive extraction (SBSE) combined with gas chromatography-mass spectrometry (GC-MS), gas chromatography-olfactometry (GC-O), odor activity value (OAV), and aroma recombination. Food Res. Int..

[25] Wang H., Ouyang W., Yu Y., Wang J., Yuan H., Hua J., Jiang Y. (2022). Analysis of non-volatile and volatile metabolites reveals the influence of second-drying heat transfer methods on green tea quality. Food Chem. X.

[26] Wang J.Q., Fu Y.Q., Chen J.X., Wang F., Feng Z.H., Yin J.F., Zeng L., Xu Y.Q. (2022). Effects of baking treatment on the sensory quality and physicochemical properties of green tea with different processing methods. Food Chem..

[27] Wang B., Qu F., Wang P., Zhao L., Wang Z., Han Y., Zhang X. (2022). Characterization analysis of flavor compounds in green teas at different drying temperature. LWT.

[28] Zeng Y., Liang J., Zhang J., Chen W., Hu D., Xia H., Ma C., Qiao X. (2023). Improvement of the sensory characteristics of Dancong tea using a dry-heating post-treatment approach. LWT.

[29] Wang H., Xie H., Chen S., Fu Q., Wang R., Zhang W., Hu Z. (2017). Effect of different drying methods on drying characteristics and qualities of lemon slices. Trans. Chin. Soc. Agric. Eng. Trans. CSAE.

[30] Luo L., Wang J., Li M., Zhang Y., Wang Y., Xu Y., Chen H., Zhu Y., Feng Z., Yin J. (2023). Characterization of the key odorants and investigation of the effects of drying methods on the aroma, taste, color and volatile profiles of the fruit of *Clausena anisum-olens* (Blanco) Merr. LWT.

[31] Peng Y., Du Z., Wang X., Wu R., Zheng C., Han W., Liu L., Gao F., Liu G., Liu B. (2024). From heat to flavor: Unlocking new chemical signatures to discriminate Wuyi rock tea under light and moderate roasting. Food Chem..

[32] Kręcisz M., Kolniak-Ostek J., Łyczko J., Stępień B. (2023). Evaluation of bioactive compounds, volatile compounds, drying process kinetics and selected physical properties of vacuum impregnation celery dried by different methods. Food Chem..

[33] Xu Y.Q., Zhang Y.N., Chen J.X., Wang F., Du Q.Z., Yin J.F. (2018). Quantitative analyses of the bitterness and astringency of catechins from green tea. Food Chem..

[34] Zhang J., Sun-Waterhouse D., Su G., Zhao M. (2019). New insight into umami receptor, umami/umami-enhancing peptides and their derivatives: A review. Trends Food Sci. Technol..

[35] Protiva Rani D., Youngmok K., Seong-Jin H., Jong-Bang E. (2019). Profiling of volatile and non-phenolic metabolites—Amino acids, organic acids, and sugars of green tea extracts obtained by different extraction techniques. Food Chem..

[36] Zeng L., Fu Y.Q., Liu Y.Y., Huang J.S., Chen J.X., Yin J.F., Jin S., Sun W.J., Xu Y.Q. (2023). Comparative analysis of different grades of Tieguanyin oolong tea based on metabolomics and sensory evaluation. LWT.

[37] Wang L.F., Kim D.M., Lee C.Y. (2000). Effects of heat processing and storage on flavanols and sensory qualities of green tea beverage. J. Agric. Food Chem..

[38] Zhang L., Xia Y., Peterson D.G. (2014). Identification of bitter modulating maillard-catechin reaction products. J. Agric. Food Chem..

[39] Guo X., Ho C.T., Schwab W., Wan X. (2021). Effect of the roasting degree on flavor quality of large-leaf yellow tea. Food Chem..

[40] Fan F.Y., Shi M., Nie Y., Zhao Y., Ye J.H., Liang Y.R. (2016). Differential behaviors of tea catechins under thermal processing: Formation of non-enzymatic oligomers. Food Chem..

[41] Jiang G., Xue R., Wang Y., Liu B., Yuan Y., Pu Q., Fang X., Huang Y., Xiang J., Hu X. (2024). Dynamic changes in the aroma profiles and volatiles of Enshi Yulu tea throughout its industrial processing. Food Chem..

[42] Zhou H., Liu Y., Yang J., Wang H., Ding Y., Lei P. (2022). Comprehensive profiling of volatile components in Taiping Houkui green tea. LWT.

[43] Yang W.Z. (2019). Understanding the biosyntheses and stress response mechanisms of aroma compounds in tea (*Camellia sinensis*) to safely and effectively improve tea aroma. Crit. Rev. Food Sci. Nutr..

[44] Ho C.T., Zheng X., Li S. (2015). Tea aroma formation. Food Sci. Hum. Wellness.

[45] Xu S., Zeng X., Wu H., Shen S., Ning J. (2021). Characterizing Volatile Metabolites in Raw Pu’er Tea Stored in Wet-Hot or Dry-Cold Environments by Performing Metabolomic Analysis and using the Molecular Sensory Science Approach. Food Chem..

[46] Wang H., Cao X., Yuan Z., Guo G. (2021). Untargeted metabolomics coupled with chemometrics approach for Xinyang Maojian green tea with cultivar, elevation and processing variations. Food Chem..

[47] Zhu Y.M., Dong J.J., Jin J., Liu J.H., Zheng X.Q., Lu J.L., Liang Y.R., Ye J.H. (2021). Roasting process shaping the chemical profile of roasted green tea and the association with aroma features. Food Chem..

[48] Xu M., Wang J., Zhu L. (2021). Tea quality evaluation by applying E-nose combined with chemometrics methods. J. Food Sci. Technol..

[49] Chen L., Wu J.E., Li Z., Liu Q., Zhao X., Yang H. (2019). Metabolomic analysis of energy regulated germination and sprouting of organic mung bean (*Vigna radiata*) using NMR spectroscopy. Food Chem..

[50] Wang Y.S., Fang M.Z., Zheng S.D., Cho J.G., Yi T.H. (2021). Identification of Chinese green tea (*Camellia sinensis*) marker metabolites using GC/MS and UPLC-QTOF/MS. Food Sci. Biotechnol..

[51] Li H., Luo L., Ma M., Zeng L. (2018). Characterization of Volatile Compounds and Sensory Analysis of Jasmine Scented Black Tea Produced by Different Scenting Processes. J. Food Sci..

[52] Yu X., Li Y., He C., Zhou J., Chen Y., Yu Z., Wang P., Ni D. (2020). Nonvolatile metabolism in postharvest tea (*Camellia sinensis* L.) leaves: Effects of different withering treatments on nonvolatile metabolites, gene expression levels, and enzyme activity. Food Chem..

[53] Wu Y., Lv S., Lian M., Wang C., Gao X., Meng Q. (2016). Study of characteristic aroma components of baked Wujiatai green tea by HS-SPME/GC-MS combined with principal component analysis. CyTA J. Food.

[54] Schuh C., Schieberle P. (2006). Characterization of the key aroma compounds in the beverage prepared from Darjeeling black tea: Quantitative differences between tea leaves and infusion. J. Agric. Food Chem..

[55] Qiao X., Zhang S., He S., Liu S. (2023). Urate oxidase treatment increases the quality of autumn Yellowish Yinghong 9 black tea. LWT.

[56] Yin X., Xiao Y., Wang K., Wu W., Huang J., Liu S., Zhang S. (2023). Effect of shaking manners on floral aroma quality and identification of key floral-aroma-active compounds in Hunan black tea. Food Res. Int..

[57] Huang D., Li M., Wang H., Fu M., Hu S., Wan X., Wang Z., Chen Q. (2023). Combining gas chromatography-ion mobility spectrometry and olfactory analysis to reveal the effect of filled-N2 anaerobic treatment duration on variation in the volatile profiles of gabaron green tea. LWT.

[58] Wang Z., Jin Q., Jiang R., Liu Y., Xie H., Ou X., Li Q., Liu Z., Huang J.A. (2024). Characteristic volatiles of Fu brick tea formed primarily by extracellular enzymes during *Aspergillus cristatus* fermentation. Food Res. Int..

[59] Xu J., Zhang Y., Hu C., Yu B., Wan C., Chen B., Lu L., Yuan L., Wu Z., Chen H. (2024). The flavor substances changes in Fuliang green tea during storage monitoring by GC–MS and GC-IMS. Food Chem. X.

[60] Tu Z., Li S., Tao M., He W., Shu Z., Wang S., Liu Z. (2025). Effect of shaking and piling processing on improving the aroma quality of green tea. Food Res. Int..

